# Supplementary figures and images for: Citrate-modified bacterial cellulose as a potential scaffolding material for bone tissue regeneration
Source: PLoS One. 2024 Dec 31;19(12):e0312396. doi: 10.1371/journal.pone.0312396 (PMC11687737; doi:10.1371/journal.pone.0312396)

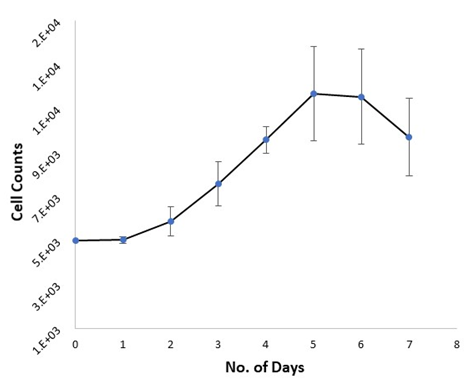


**S1 Fig. Average growth curve of hFOB cell lines cultured on CDMEM.**

Supplement: S1 Fig — (DOCX) [file pone.0312396.s001.docx]
