# Supplementary material for: Citrate-modified bacterial cellulose as a potential scaffolding material for bone tissue regeneration
Source: PLoS One. 2024 Dec 31;19(12):e0312396. doi: 10.1371/journal.pone.0312396 (PMC11687737; doi:10.1371/journal.pone.0312396)
Supplement: S1 Table — (DOCX) [file pone.0312396.s002.docx]

**S1 Table. Cell initial seeding density and corresponding % confluency/day.**

| **S/N** | **Initial seeding density** | **% Confluency/day** | | |
| --- | --- | --- | --- | --- |
|  |  | **1** | **2** | **3** |
| **1** | 5x10^4^ | 30 | 60 | 90 |
| **2** | 1x10^5^ | 70 | 90 | 100 |
| **3** | 2x105 | 90 | 100 | 100+ |
| **4** | 3x10^5^ | 100 | 100+ | 100+ |
| **5** | 4x10^5^ | 100+ | 100+ | 100+ |
| **6** | 5x10^5^ | 100+ | 100+ | 100+ |
