# Supplementary material for: Citrate-modified bacterial cellulose as a potential scaffolding material for bone tissue regeneration
Source: PLoS One. 2024 Dec 31;19(12):e0312396. doi: 10.1371/journal.pone.0312396 (PMC11687737; doi:10.1371/journal.pone.0312396)
Supplement: S2 Table — (DOCX) [file pone.0312396.s003.docx]

**S2 Table. One-way ANOVA and Turkey Post Hoc Multiple comparisons test for 3 days MTS assay**

| Input | | | N of Rows in Working Data File | | | | | | 30 | | | | |
| --- | --- | --- | --- | --- | --- | --- | --- | --- | --- | --- | --- | --- | --- |
| Syntax | | | | | | | | | One-way OD3 by group  Post Hoc = Tukey Alpha (0.05). | | | | |
| **ANOVA** | | | | | | | | | | | | | |
| OD3 | | | | | | | | | | | | | |
|  | | Sum of Squares | | | df | | Mean Square | | | | F | | Sig. |
| Between Groups | | .417 | | | 4 | | .104 | | | | 3.114 | | .033 |
| Within Groups | | .837 | | | 25 | | .033 | | | |  | |  |
| Total | | 1.254 | | | 29 | |  | | | |  | |  |
| **Post Hoc Tests Multiple Comparisons** | | | | | | | | | | | | | |
| Dependent Variable: OD3 | | | | | | | | | | | | | |
| Tukey HSD | | | | | | | | | | | | | |
| (I) group | (J) group | | | Mean Difference (I-J) | | Std. Error | | Sig. | | 95% Confidence Interval | | | |
|  |  |  |  |  |  |  |  |  |  | Lower Bound | | Upper Bound | |
| control | BC | | | .07487 | | .10565 | | .953 | | -.2354 | | .3851 | |
|  | BC-S2 | | | .12220 | | .10565 | | .775 | | -.1881 | | .4325 | |
|  | BMBC0.03-S2 | | | .32737^*^ | | .10565 | | .035 | | .0171 | | .6376 | |
|  | BMBC0.07-S2 | | | .24603 | | .10565 | | .169 | | -.0642 | | .5563 | |
| BC | control | | | -.07487 | | .10565 | | .953 | | -.3851 | | .2354 | |
|  | BC-S2 | | | .04733 | | .10565 | | .991 | | -.2629 | | .3576 | |
|  | BMBC0.03-S2 | | | .25250 | | .10565 | | .151 | | -.0578 | | .5628 | |
|  | BMBC0.07-S2 | | | .17117 | | .10565 | | .499 | | -.1391 | | .4814 | |
| BC-S2 | control | | | -.12220 | | .10565 | | .775 | | -.4325 | | .1881 | |
|  | BC | | | -.04733 | | .10565 | | .991 | | -.3576 | | .2629 | |
|  | BMBC0.03-S2 | | | .20517 | | .10565 | | .322 | | -.1051 | | .5154 | |
|  | BMBC0.07-S2 | | | .12383 | | .10565 | | .767 | | -.1864 | | .4341 | |
| BMBC0.03-S2 | control | | | -.32737^*^ | | .10565 | | .035 | | -.6376 | | -.0171 | |
|  | BC | | | -.25250 | | .10565 | | .151 | | -.5628 | | .0578 | |
|  | BC-S2 | | | -.20517 | | .10565 | | .322 | | -.5154 | | .1051 | |
|  | BMBC0.07-S2 | | | -.08133 | | .10565 | | .937 | | -.3916 | | .2289 | |
| BMBC0.07-S2 | control | | | -.24603 | | .10565 | | .169 | | -.5563 | | .0642 | |
|  | BC | | | -.17117 | | .10565 | | .499 | | -.4814 | | .1391 | |
|  | BC-S2 | | | -.12383 | | .10565 | | .767 | | -.4341 | | .1864 | |
|  | BMBC0.03-S2 | | | .08133 | | .10565 | | .937 | | -.2289 | | .3916 | |
| *. The mean difference is significant at the 0.05 level. | | | | | | | | | | | | | |

**Homogeneous Subsets**

| **OD3** | | | |
| --- | --- | --- | --- |
| Tukey HSD^a^ | | | |
| group | N | Subset for alpha = 0.05 | |
|  |  | 1 | 2 |
| BMBC0.03-S2 | 6 | 2.2352 |  |
| BMBC0.07-S2 | 6 | 2.3165 | 2.3165 |
| BC-S2 | 6 | 2.4403 | 2.4403 |
| BC | 6 | 2.4877 | 2.4877 |
| control | 6 |  | 2.5625 |
| Sig. |  | .151 | .169 |
| Means for groups in homogeneous subsets are displayed. | | | |
| a. Uses Harmonic Mean Sample Size = 6.000. | | | |
