# Supplementary material for: Citrate-modified bacterial cellulose as a potential scaffolding material for bone tissue regeneration
Source: PLoS One. 2024 Dec 31;19(12):e0312396. doi: 10.1371/journal.pone.0312396 (PMC11687737; doi:10.1371/journal.pone.0312396)
Supplement: S3 Table — (DOCX) [file pone.0312396.s004.docx]

**S3 Table. One-way ANOVA and Turkey Post Hoc Multiple comparisons test for 5 days MTS assay**

| Input | | No. of Rows in Working Data File | | | | | | 30 | | | | |
| --- | --- | --- | --- | --- | --- | --- | --- | --- | --- | --- | --- | --- |
| Syntax | | | | | | | | One-way OD5 by group  Post Hoc = Tukey Alpha (0.05). | | | | |
| **ANOVA** | | | | | | | | | | | | |
| OD5 | | | | | | | | | | | | |
|  | | | Sum of Squares | | df | | Mean Square | | | F | | Sig. |
| Between Groups | | | .531 | | 4 | | .133 | | | .525 | | .719 |
| Within Groups | | | 6.325 | | 25 | | .253 | | |  | |  |
| Total | | | 6.856 | | 29 | |  | | |  | |  |
| **Post Hoc Tests Multiple Comparisons** | | | | | | | | | | | | |
| Dependent Variable: OD5 | | | | | | | | | | | | |
| Tukey HSD | | | | | | | | | | | | |
| (I) group | (J) group | | | Mean Difference (I-J) | | Std. Error | | Sig. | 95% Confidence Interval | | | |
|  |  |  |  |  |  |  |  |  | Lower Bound | | Upper Bound | |
| control | BC | | | .12867 | | .29041 | | .992 | -.7242 | | .9816 | |
|  | BC-S2 | | | .17017 | | .29041 | | .976 | -.6827 | | 1.0231 | |
|  | BMBC0.03-S2 | | | .20550 | | .29041 | | .953 | -.6474 | | 1.0584 | |
|  | BMBC0.07-S2 | | | .40967 | | .29041 | | .627 | -.4432 | | 1.2626 | |
| BC | control | | | -.12867 | | .29041 | | .992 | -.9816 | | .7242 | |
|  | BC-S2 | | | .04150 | | .29041 | | 1.000 | -.8114 | | .8944 | |
|  | BMBC0.03-S2 | | | .07683 | | .29041 | | .999 | -.7761 | | .9297 | |
|  | BMBC0.07-S2 | | | .28100 | | .29041 | | .867 | -.5719 | | 1.1339 | |
| BC-S2 | control | | | -.17017 | | .29041 | | .976 | -1.0231 | | .6827 | |
|  | BC | | | -.04150 | | .29041 | | 1.000 | -.8944 | | .8114 | |
|  | BMBC0.03-S2 | | | .03533 | | .29041 | | 1.000 | -.8176 | | .8882 | |
|  | BMBC0.07-S2 | | | .23950 | | .29041 | | .920 | -.6134 | | 1.0924 | |
| BMBC0.03-S2 | control | | | -.20550 | | .29041 | | .953 | -1.0584 | | .6474 | |
|  | BC | | | -.07683 | | .29041 | | .999 | -.9297 | | .7761 | |
|  | BC-S2 | | | -.03533 | | .29041 | | 1.000 | -.8882 | | .8176 | |
|  | BMBC0.07-S2 | | | .20417 | | .29041 | | .954 | -.6487 | | 1.0571 | |
| BMBC0.07-S2 | control | | | -.40967 | | .29041 | | .627 | -1.2626 | | .4432 | |
|  | BC | | | -.28100 | | .29041 | | .867 | -1.1339 | | .5719 | |
|  | BC-S2 | | | -.23950 | | .29041 | | .920 | -1.0924 | | .6134 | |
|  | BMBC0.03-S2 | | | -.20417 | | .29041 | | .954 | -1.0571 | | .6487 | |

**Homogeneous Subsets**

| **OD5** | | |
| --- | --- | --- |
| Tukey HSD^a^ | | |
| group | N | Subset for alpha = 0.05 |
|  |  | 1 |
| BMBC0.07-S2 | 6 | 3.0282 |
| BMBC0.03-S2 | 6 | 3.2323 |
| BC-S2 | 6 | 3.2677 |
| BC | 6 | 3.3092 |
| control | 6 | 3.4378 |
| Sig. |  | .627 |
| Means for groups in homogeneous subsets are displayed. | | |
| a. Uses Harmonic Mean Sample Size = 6.000. | | |
