# Supplementary material for: Citrate-modified bacterial cellulose as a potential scaffolding material for bone tissue regeneration
Source: PLoS One. 2024 Dec 31;19(12):e0312396. doi: 10.1371/journal.pone.0312396 (PMC11687737; doi:10.1371/journal.pone.0312396)
Supplement: S4 Table — (DOCX) [file pone.0312396.s005.docx]

S4 Table. One-way ANOVA and Turkey Post Hoc Multiple comparisons test for 7 days MTS assay

| Input | | No. of Rows in Working Data File | | | | | | 30 | | | | |
| --- | --- | --- | --- | --- | --- | --- | --- | --- | --- | --- | --- | --- |
| Syntax | | | | | | | | One-way OD7 by group  Post Hoc = Tukey Alpha (0.05). | | | | |
| **ANOVA** | | | | | | | | | | | | |
| OD7 | | | | | | | | | | | | |
|  | | | Sum of Squares | | df | | Mean Square | | | F | | Sig. |
| Between Groups | | | .434 | | 4 | | .109 | | | .405 | | .804 |
| Within Groups | | | 6.705 | | 25 | | .268 | | |  | |  |
| Total | | | 7.139 | | 29 | |  | | |  | |  |
| **Post Hoc Tests Multiple Comparisons** | | | | | | | | | | | | |
| Dependent Variable: OD7 | | | | | | | | | | | | |
| Tukey HSD | | | | | | | | | | | | |
| (I) group | (J) group | | | Mean Difference (I-J) | | Std. Error | | Sig. | 95% Confidence Interval | | | |
|  |  |  |  |  |  |  |  |  | Lower Bound | | Upper Bound | |
| Control | BC | | | .19080 | | .29901 | | .967 | -.6873 | | 1.0689 | |
|  | BC-S2 | | | .06563 | | .29901 | | .999 | -.8125 | | .9438 | |
|  | BMBC0.03-S2 | | | .27630 | | .29901 | | .885 | -.6018 | | 1.1544 | |
|  | BMBC0.07-S2 | | | .31430 | | .29901 | | .829 | -.5638 | | 1.1924 | |
| BC | control | | | -.19080 | | .29901 | | .967 | -1.0689 | | .6873 | |
|  | BC-S2 | | | -.12517 | | .29901 | | .993 | -1.0033 | | .7530 | |
|  | BMBC0.03-S2 | | | .08550 | | .29901 | | .998 | -.7926 | | .9636 | |
|  | BMBC0.07-S2 | | | .12350 | | .29901 | | .994 | -.7546 | | 1.0016 | |
| BC-S2 | control | | | -.06563 | | .29901 | | .999 | -.9438 | | .8125 | |
|  | BC | | | .12517 | | .29901 | | .993 | -.7530 | | 1.0033 | |
|  | BMBC0.03-S2 | | | .21067 | | .29901 | | .954 | -.6675 | | 1.0888 | |
|  | BMBC0.07-S2 | | | .24867 | | .29901 | | .918 | -.6295 | | 1.1268 | |
| BMBC0.03-S2 | control | | | -.27630 | | .29901 | | .885 | -1.1544 | | .6018 | |
|  | BC | | | -.08550 | | .29901 | | .998 | -.9636 | | .7926 | |
|  | BC-S2 | | | -.21067 | | .29901 | | .954 | -1.0888 | | .6675 | |
|  | BMBC0.07-S2 | | | .03800 | | .29901 | | 1.000 | -.8401 | | .9161 | |
| BMBC0.07-S2 | control | | | -.31430 | | .29901 | | .829 | -1.1924 | | .5638 | |
|  | BC | | | -.12350 | | .29901 | | .994 | -1.0016 | | .7546 | |
|  | BC-S2 | | | -.24867 | | .29901 | | .918 | -1.1268 | | .6295 | |
|  | BMBC0.03-S2 | | | -.03800 | | .29901 | | 1.000 | -.9161 | | .8401 | |

**Homogeneous Subsets**

| **OD7** | | |
| --- | --- | --- |
| Tukey HSD^a^ | | |
| group | N | Subset for alpha = 0.05 |
|  |  | 1 |
| BMBC0.07-S2 | 6 | 4.1923 |
| BMBC0.03-S2 | 6 | 4.2303 |
| BC | 6 | 4.3158 |
| BC-S2 | 6 | 4.4410 |
| control | 6 | 4.5066 |
| Sig. |  | .829 |
| Means for groups in homogeneous subsets are displayed. | | |
| a. Uses Harmonic Mean Sample Size = 6.000. | | |
