# Supplementary material for: Citrate-modified bacterial cellulose as a potential scaffolding material for bone tissue regeneration
Source: PLoS One. 2024 Dec 31;19(12):e0312396. doi: 10.1371/journal.pone.0312396 (PMC11687737; doi:10.1371/journal.pone.0312396)
Supplement: S5 Table — (DOCX) [file pone.0312396.s006.docx]

**S5 Table. One-way ANOVA and Turkey Post Hoc Multiple comparisons test between groups (3, 5, and 7 days) MTS assay**

| Input | | | | N of Rows in Working Data File | | | | 18 | | | | |
| --- | --- | --- | --- | --- | --- | --- | --- | --- | --- | --- | --- | --- |
| Syntax | | | | | | | | One-way All Groups by days  Post Hoc = Tukey Alpha (0.05). | | | | |
| Resources | | | | Processor Time | | | | 00:00:00.03 | | | | |
|  |  |  |  | Elapsed Time | | | | 00:00:00.02 | | | | |
| **ANOVA** | | | | | | | | | | | | |
| All Groups | | | | | | | | | | | | |
|  | | Sum of Squares | | | df | | Mean Square | | | F | | Sig. |
| Between Groups | | 10.061 | | | 2 | | 5.030 | | | 41.013 | | .000 |
| Within Groups | | 1.840 | | | 15 | | .123 | | |  | |  |
| Total | | 11.901 | | | 17 | |  | | |  | |  |
| **Post Hoc Tests Multiple Comparisons** | | | | | | | | | | | | |
| Dependent Variable: All Groups | | | | | | | | | | | | |
| Tukey HSD | | | | | | | | | | | | |
| (I) DAYS | (J) DAYS | | Mean Difference (I-J) | | Std. Error | Sig. | | | 95% Confidence Interval | | | |
|  |  |  |  |  |  |  |  |  | Lower Bound | | Upper Bound | |
| 3 days | 5 days | | -.82150^*^ | | .20220 | .003 | | | -1.3467 | | -.2963 | |
|  | 7 days | | -1.82817^*^ | | .20220 | .000 | | | -2.3534 | | -1.3030 | |
| 5 days | 3 days | | .82150^*^ | | .20220 | .003 | | | .2963 | | 1.3467 | |
|  | 7 days | | -1.00667^*^ | | .20220 | .000 | | | -1.5319 | | -.4815 | |
| 7 days | 3 days | | 1.82817^*^ | | .20220 | .000 | | | 1.3030 | | 2.3534 | |
|  | 5 days | | 1.00667^*^ | | .20220 | .000 | | | .4815 | | 1.5319 | |
| *. The mean difference is significant at the 0.05 level. | | | | | | | | | | | | |

**Homogeneous Subsets**

| **All groups** | | | | |
| --- | --- | --- | --- | --- |
| Tukey HSD^a^ | | | | |
| DAYS | N | Subset for alpha = 0.05 | | |
|  |  | 1 | 2 | 3 |
| 3 days | 6 | 2.4877 |  |  |
| 5 days | 6 |  | 3.3092 |  |
| 7 days | 6 |  |  | 4.3158 |
| Sig. |  | 1.000 | 1.000 | 1.000 |
| Means for groups in homogeneous subsets are displayed. | | | | |
| a. Uses Harmonic Mean Sample Size = 6.000. | | | | |
