# Supplementary material for: Citrate-modified bacterial cellulose as a potential scaffolding material for bone tissue regeneration
Source: PLoS One. 2024 Dec 31;19(12):e0312396. doi: 10.1371/journal.pone.0312396 (PMC11687737; doi:10.1371/journal.pone.0312396)
Supplement: Attachment — Submitted filename: Response to Reviewers.docx [file pone.0312396.s007.docx]

**RESPONSE TO REVIEWERS**

Dear prof. Katriina Aalto-Setala

Editor in Chief.

Dear Sir/Madam,

We are grateful to the editors and reviewers for their time and constructive comments on our manuscript, PONE-D-24-23106, entitled **“citrate-modified bacterial cellulose as a potential scaffolding material for bone tissue regeneration.”** We have implemented the comments and suggestions and wish to resubmit a revised version of the manuscript for further consideration in the journal as earlier advised by the editor. We provide in tabular form a point-by-point response explaining how we have addressed the reviewers’ comments below.

We look forward to the outcome of your assessment.

Yours sincerely,

On behalf of the authors

Dr. Rabiu Salihu

[Salihu.r@fud.edu.ng](mailto:Salihu.r@fud.edu.ng)

**Reviewer 1:**

The authors present results regarding the synthesis of the HA crystals and the modified BC (MBC) samples through the SBF immersion method. The manuscripts present the characterization of the biomineralized MBC (BMBC) samples by attenuated total reflectance Fourier transform infrared (ATR-FTIR) spectroscopy, X-ray diffraction (XRD), Field emission scanning electron microscopy (FE-SEM), and thermal gravimetric analysis (TGA). Also, the authors provided information on the biocompatibility of the BMBC to human osteoblast cell lines. The biocompatibility of the samples was evaluated through the MTS, Trypan blue dye exclusion (TBDE), and attachment assays.

The overall manuscript is well structured and present useful information about the topic.

Nonetheless, the manuscript needs major improvements before being considered for publication. Please have in mind the following improvements:

| **S/N** | **Comments** | **Rebuttal/Response** |
| --- | --- | --- |
| **1.** | The abstract should be more concise and state the motivation and the major achievements of the study. | This has been addressed by reviewing the abstract to capture both the motivation and the major achievements of the study in a concise manner. |
| **2.** | The introduction should be improved, and the authors should emphasize the novelty of their study compared to existing studies. | The authors have attempted improving the introduction part to emphasize the novelty of the study. |
| **3.** | Also, the author should add the EDX spectra to the manuscript as well as the images of hFOB 1.19 cells cultured on CDMEM which were provided in the supplementary section. | Both the EDX and the images of hFOB 1.19 cells with brief explanations were added to the manuscript accordingly. |
| **4.** | The FTIR vibrational bands should be assigned and summarized in a table in the manuscript. Also, the second derivative of the FTIR should be included. | The table for the FTIR vibrational bands as well as the second derivatives was not included with the intention to comply with conventional practice of not repeating in the table what is already in the text. Moreover, the fact that the work is based on our previous report (reference [37] in the manuscript), the major characterizations of the modified bacterial cellulose were presented. Therefore, the emphasis was only on the peaks associated with the HA in the BC; however, the corresponding wavenumbers have been assigned to the major differing bands. |
| **5.** | The discussions section should be improved. The biological assays should be correlated with the physico-chemical properties of the samples. Also, the authors should provide a possible explanation regarding why according to the in-vitro biocompatibility assessment, the modified and biomineralized samples have demonstrated better cell proliferation and attachment compared to the control. | The authors have attempted to improve the discussion section to relate the biological assays with physico-chemical properties and offer explanations on the better cell proliferation and attachment displayed by modified and biomineralized samples compared to controls. |
| **6.** | The conclusions section should also be improved. | The conclusion section has also been improved by the authors. |

**Reviewer 2:**

| **S/N** | **Comments** | **Rebuttal/Response** |
| --- | --- | --- |
| **1.** | It is necessary to edit the native English language. | The authors have sent the manuscript for grammar checking, and we believe that major grammatical errors have been taken care of. |
| **2.** | The image quality should be enhanced. | This has been addressed in compliance with the journals’ formatting guidelines. |
| **3.** | The manuscript does not mention the innovative aspects of the work. | This has been addressed by carefully reviewing and making the innovation clearer in many parts of the manuscript. |
| **4.** | Please revise the discussion section to include the limitations and suggestions. Additionally, highlight the advantages of your work compared to other materials. | The authors have attempted improving the discussion section to include the limitations and suggestions highlighted by the reviewer. |
| **5.** | Bacterial cellulose has a significantly small pore diameter. This raises the question of how it can serve as a suitable biomaterial for bone tissue engineering. The challenge lies in the inability to efficiently transfer cells to the central layers. please discussion about this limitation. | The authors have attempted addressing this concern with reference to previously published articles. |
